# A pyrexic effect of FGF21 independent of energy expenditure and UCP1

**DOI:** 10.1016/j.molmet.2021.101324

**Published:** 2021-08-19

**Authors:** Petr Zouhar, Petra Janovska, Sara Stanic, Kristina Bardova, Jiri Funda, Blanka Haberlova, Birgitte Andersen, Martin Rossmeisl, Barbara Cannon, Jan Kopecky, Jan Nedergaard

**Affiliations:** 1 Laboratory of Adipose Tissue Biology, Institute of Physiology of the Czech Academy of Sciences, Prague, Czech Republic; 2 Global Drug Discovery, Novo Nordisk A/S, Måløv, Denmark; 3 Department of Molecular Biosciences, The Wenner-Gren Institute, Stockholm University, Stockholm, Sweden

**Keywords:** UCP1, Thermoneutrality, Beiging/browning, Obesity, Body temperature control

## Abstract

**Objective:**

Administration of FGF21 to mice reduces body weight and increases body temperature. The increase in body temperature is generally interpreted as hyperthermia, i.e. a condition secondary to the increase in energy expenditure (heat production). Here, we examine an alternative hypothesis: that FGF21 has a direct pyrexic effect, i.e. FGF21 increases body temperature independently of any effect on energy expenditure.

**Methods:**

We studied the effects of FGF21 treatment on body temperature and energy expenditure in high-fat-diet-fed and chow-fed mice exposed acutely to various ambient temperatures, in high-fat diet-fed mice housed at 30 °C (i.e. at thermoneutrality), and in mice lacking uncoupling protein 1 (UCP1).

**Results:**

In every model studied, FGF21 increased body temperature, but energy expenditure was increased only in some models. The effect of FGF21 on body temperature was more (not less, as expected in hyperthermia) pronounced at lower ambient temperatures. Effects on body temperature and energy expenditure were temporally distinct (daytime versus nighttime). FGF21 enhanced UCP1 protein content in brown adipose tissue (BAT); there was no measurable UCP1 protein in inguinal brite/beige adipose tissue. FGF21 increased energy expenditure through adrenergic stimulation of BAT. In mice lacking UCP1, FGF21 did not increase energy expenditure but increased body temperature by reducing heat loss, e.g. a reduced tail surface temperature.

**Conclusion:**

The effect of FGF21 on body temperature is independent of UCP1 and can be achieved in the absence of any change in energy expenditure. Since elevated body temperature is a primary effect of FGF21 and can be achieved without increasing energy expenditure, only limited body weight-lowering effects of FGF21 may be expected.

## Highlights

• FGF21 increases body temperature independently of energy expenditure changes.
• FGF21-induced increase in body temperature is independent of UCP1.
• FGF21-induced (short-term) increase in energy expenditure is fully UCP1-dependent.
• FGF21 increases UCP1 content in brown, but not in brite/beige adipose tissue.
• FGF21 can increase body temperature by decreasing heat loss.

## Graphical abstract

**Figure:**
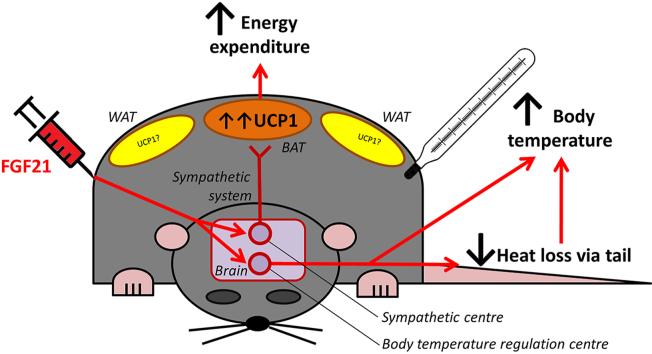

## 1 Introduction

Fibroblast growth factor 21 (**FGF21**) is a hormone secreted in response to several stress stimuli, such as cold, fasting, or a ketogenic diet [1]. The physiologic role of FGF21 is still under debate, but pharmacologic administration of FGF21 to laboratory rodents has several beneficial effects on insulin sensitivity and energy metabolism. Particularly, FGF21 treatment is reported to lower body weight, presumably by induction of increased energy expenditure [[2], [3], [4], [5]]. This increase in energy expenditure has been thought to be due to induction of uncoupling protein 1 (**UCP1**) – and thus nonshivering thermogenesis – in brown adipose tissue (**BAT**), and especially in white/brite/beige adipose tissue (**WAT**) [[2], [3], [4], [5], [6]].

FGF21 treatment also increases body temperature. This increase has until now been interpreted as a consequence of the increased energy expenditure (heat production) [5], implying that the increase is hyperthermia. The FGF21-treated mice are thought to produce more heat than the mice can dissipate, and therefore overheat. Correspondingly, Fisher et al. [7] observed that FGF21-deficient mice maintained a lower body temperature than control mice when they were chronically exposed to 5 °C. The authors interpreted this lower body temperature as an indication that thermogenesis was lower in the FGF21-deficient mice and was thus insufficient to defend body temperature, i.e. the FGF21-deficient mice were considered to be hypothermic – unable to produce sufficient heat to counteract heat loss. This implied that FGF21 is essential for the development of full thermogenic capacity in the cold.

In contrast to the belief that FGF21 treatment increases energy expenditure and thus causes hyperthermia, we report here an alternative explanation: FGF21 increases body temperature directly by elevating the body temperature “set-point” in the hypothalamus (while an absence of FGF21 would decrease this body temperature “set point”). We use the term set-point pragmatically for simplicity [8]. Thus, we hypothesize that FGF21 induces “fever” (pyrexia).

Experiments addressing questions of energy expenditure with a translational perspective should be conducted at thermoneutral temperature, i.e. the temperature at which the basal metabolism of the subject is sufficient to maintain stable body temperature without any need for additional thermogenesis [9]. While the thermoneutral temperature for lightly dressed humans is about 20 °C, for normal adult mice it is close to 30 °C [9,10]. With very few exceptions [6], earlier experiments investigating thermogenic and body weight-lowering effects of FGF21 have been conducted at sub-thermoneutral, standard animal house temperatures of 20–24 (26) °C (e.g. [[2], [3], [4], [5],^11^]), complicating the translation of the results to humans. The results presented here are all based on mice acclimated to thermoneutrality.

Elucidating the mechanism of FGF21 action (i.e. whether FGF21 causes hyperthermia following increased energy expenditure or whether it causes an increase in body temperature setpoint, i.e. pyrexia) is of considerable translational importance since the induction of energy expenditure is the goal of body weight-lowering strategies. If the primary effect of FGF21 is an increase in body temperature setpoint rather than an increase in energy expenditure, the translational perspectives of FGF21 treatment as an anti-obesity strategy would be less promising.

## 2 Methods

### 2.1 Experimental design

The presented data were obtained in four independent experiments described below. Mice were housed in the animal facility of the Institute of Physiology, Czech Academy of Sciences, on a 12:12 light: dark cycle (light on at 6 am) with free access to water and food. During the experiments, animals were single-caged and housed at a thermoneutral temperature of 30 °C, unless otherwise stated. All experiments were approved by the Animal Care and Use Committee of the Institute of Physiology of the Czech Academy of Sciences (Approval Number 81/2016) and followed the guidelines.

#### 2.1.1 Experiment 1 (see also the scheme in Figure 1A)

The experiment was performed on 18-week old C57BL/6J male mice (Taconic). Prior to the start of the study (day 0), the mice had been fed for 10 weeks either standard chow pellets (RM-H extruded diet, Ssniff, Germany; metabolizable energy: 13.9 MJ/kg; 10% energy in form of fat), or high-fat diet pellets (**HFD**; E15742-34, Ssniff; metabolizable energy: 21.4 MJ/kg; 60% energy in the form of fat). They were single-caged 2 weeks before the start of the study and habituated to thermoneutrality by housing at 30 °C for 2–3 weeks (HFD-fed mice) or 9–10 weeks (chow-fed mice), before day 0.

**Figure 1:**
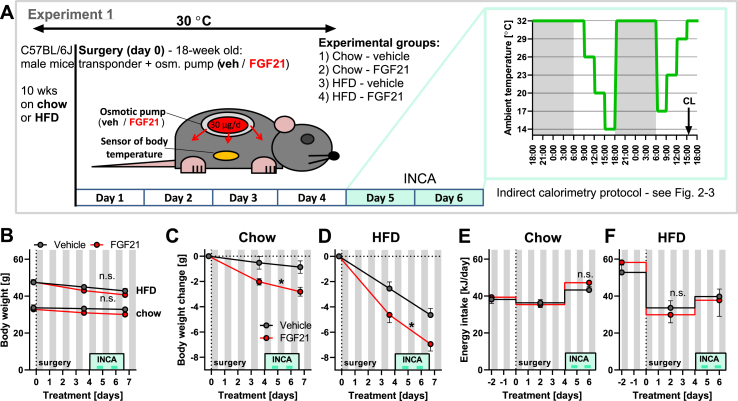
**Effects of FGF21 on body weight and energy intake.** Experiment 1: C57BL/6J mice on chow or HFD with 30 μg/d FGF21 or vehicle delivered by osmotic minipump for 5–7 days (n = 4–8). The same mice as shown in Figure 2 and part of Figure 3. [A] Scheme of the experiment including the INCA measurements on days 4 and 5 when mice were exposed to various ambient temperatures. See Methods for further details. [B] Development of body weight during the treatment. Grey rectangles in this and similar graphs represent the dark period of the day, light green rectangles represent the time when mice were placed in the INCA chamber, and the darker green lines represent the time of measurements shown in Figure 2. [C-D] Body weight change of mice fed by chow (C) and HFD (D) during the treatment. Body weight immediately after the surgery is set to 0. [E-F] Energy intake of mice fed chow (E) and HFD (F) during the treatment. Statistics: Two-way repeated measures ANOVA (B–E) or mixed-effects model (REML, as some individual values are missing because of food spilling (F)). ∗ represents a significant difference (p < 0.05) between FGF21 and vehicle treatments (comparing whole curves).

On day 0, the mice were implanted i.p. with telemetry transponders (Minimitter, Respironics; to measure core body temperature and physical activity), and s.c. (in the dorsal posterior area) with osmotic minipumps (2002, Alzet) filled either with human recombinant FGF21 (delivery rate of 30 μg/d FGF21, roughly comparable with [11,12]; endotoxin level <0.07 EU/mg); or its vehicle (10 mM phosphate, 20 mg/ml glycerol, pH 7.6). In this experiment, the presumably stable FGF21 blood level achieved using minipumps allowed for a total daily dose of FGF21 lower than that used in experiments 2–4, in which the FGF21 was delivered by subcutaneous injections.

FGF21 delivery was validated by post-mortem measurement of plasma human FGF21 using FGF21 Human ELISA, BioVendor: vehicle- and FGF21-treated mice had <500 and >50,000 pg/ml FGF21, respectively.

On day 4, mice were placed in the indirect calorimetry chamber (**INCA**, Somedic) and their energy expenditure, body temperature, and physical activity were assessed at various temperatures, according to a modified protocol [13]: the mice were exposed to 32 °C (6 pm - 9 am), 26 °C (9 am - 12 pm), 20 °C (12 - 3 pm), 14 °C (3–6 pm), 32 °C (6 pm–6 am), 17 °C (6–9 am), 23 °C (9 am–12 pm), and 28 °C (12–3 pm). Thereafter, the temperature was returned to 32 °C, and the mice were injected with 35 μg CL 316,243 (β_3_ adrenergic agonist) and 3 h later euthanized by carotid bleeding under ether anaesthesia. Inguinal WAT and interscapular BAT samples were collected and frozen in liquid nitrogen for immunoblots.

#### 2.1.2 Experiment 2 (see also the scheme in Figure 3E,F)

C57BL/6J mice (own breeding facility) on HFD diet (same as in Experiment 1) and adapted to 30 °C, were implanted with transponders (as above). Two weeks later (at age 33 weeks), they were placed in INCA chambers set to a temperature of 30 °C (day 1) and allowed to habituate to the new environment for 3 days. On day 4, at ≈10 am, the mice were injected i.p. either with 350 μg propranolol or with its vehicle (phosphate-buffered saline, PBS) (n = 3). After an hour (11 am), all mice were injected with 30 μg CL 316,243. Two hours later (at 1 pm), the mice which first received PBS were injected with propranolol, to achieve a comparable endpoint in all mice. The body temperature and energy expenditure elevated originally by CL 316,243 were fully normalized at the end of day 5.

**Figure 3:**
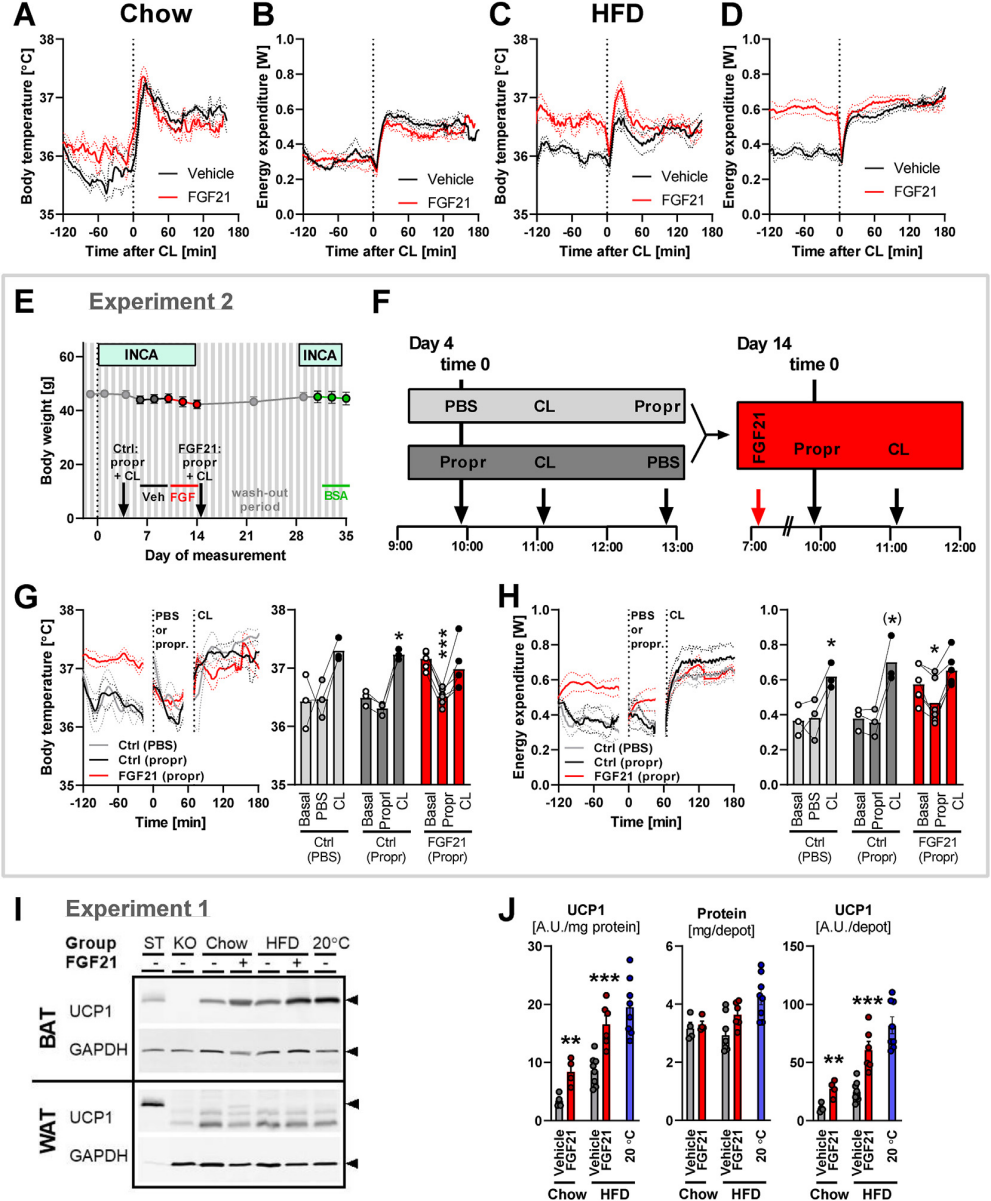
**Effects of FGF21 on thermogenic capacity and UCP1 content.** Experiment 1: The same mice as in Figure 1, Figure 2. Experiment 2: C57BL/6J mice on HFD treated twice daily for 4 days with s.c. injections of vehicle, then with 45 μg FGF21, and eventually with 45 μg bovine albumin. The same mice as in Figure 4 and part of Figure 8. [A-B] Body temperature (A) and energy expenditure (B) before and after injection of CL 316,243 (CL) in chow-fed mice. Note that all mice received an FGF21 or vehicle injection approximately 60 min prior to CL 316,243 injection, which may have affected the basal values. [C-D] Body temperature (C) and energy expenditure (D) before and after injection of CL 316,243 in HFD-fed mice. [E] Development of body weight throughout Experiment 2. The light green boxes represent the time when mice were placed in the INCA chamber. Black, red, and green lines represent the treatment with vehicle, FGF21, and bovine albumin, respectively. The black arrows represent the time of propranolol (Propr) and CL 316,243 (CL) treatment (see F). [F] Scheme of sequential treatment with propranolol (Propr) or phosphate-buffered saline (PBS) and CL 316,243 (CL) in control conditions (grey boxes, n = 3) and after FGF21 treatment (red box, n = 6). [G] Changes of body temperature during the treatment with propranolol and CL 316,243. Average curves (left panel) and statistically evaluated mean values of body temperature (right panel; Basal – mean values from time −70 to −10 min; Propr – mean values from time 10–50 min; CL – mean values from time 110–170 min). [H] Changes of energy expenditure during the treatment with propranolol and CL 316,243. Average curves (left panel) and statistically evaluated mean values of body temperature (right panel; as in G). [I] Representative immunoblots showing UCP1 and GAPDH (used as a loading control) in BAT (2 μg protein loaded per well) and WAT (20 μg protein per well) of chow- and HFD-fed mice at thermoneutrality. For comparison, samples of age-matched HFD-fed mice housed at 20 °C were included. 2 μg of the same standard (ST) BAT sample was loaded on all the blots in this and other experiments and used for normalization, making the quantified UCP1 amount comparable among the experiments. 2 μg (on the upper membrane) or 20 μg protein (on the lower membrane) of BAT from UCP1-deficient mice was used as a negative control. Black arrows on the right show the sizes of UCP1 (33 kDa) and GAPDH (37 kDa). [J] Quantification of UCP1 protein amount in interscapular BAT of chow and HFD-fed mice housed at thermoneutrality (data from a parallel group of mice housed at 20 °C are included as a reference): UCP1 expressed per unit of protein (quantification of immunoblot band intensity; left panel); total protein amount in interscapular BAT (middle panel); total UCP1 protein amount in the entire interscapular BAT depot (calculated from UCP1 per protein multiplied by total protein; right panel). Statistics: Two-way repeated measures ANOVA and Tukey's multiple comparison test for comparison of basal conditions vs. treatment with propranolol and with CL 316,243 (G–H); Student's t-test for comparison of FGF21 and vehicle treatments (J): ∗p < 0.05, ∗∗p < 0.01, ∗∗∗p < 0.001.

Subsequently, mice from both groups (i.e. propranolol + CL 316,243 and PBS + CL 316,243+propranolol) were merged in one group. Between the evening of day 6 and the morning of day 10, all mice were injected subcutaneously with vehicle twice daily (45 μl of 10 mM phosphate, 20 mg/ml glycerol, pH 7.6; at 7 am and 7 pm). Between the evening of day 10 and the morning of day 14, the mice received twice-daily subcutaneous injections of 45 μg FGF21.

On day 14, 2 h after the last dose of FGF21, the mice were again injected with 350 μg propranolol (at ≈10 am) and 30 μg CL 316,243 (at ≈11 am; Figure 3F). In this phase, the tail surface temperature of the mice was measured using an infrared camera (FLIR T860) on three occasions: before the propranolol injection, 1 h later (i.e. just before the CL 316,243 injection), lastly additional 1 h later.

Mice were then kept out of indirect calorimetry at 30 °C for 2 weeks as a wash-out and then placed in indirect calorimetry chambers again (day 29). Between the evening of day 31 and the morning of day 35, the mice received twice-daily subcutaneous injections of 45 μg bovine serum albumin. The effect of FGF21 treatment (days 10–14) was then compared to the effect of vehicle treatment (days 6–10) and of albumin treatment (days 31–35).

#### 2.1.3 Experiment 3 (see also a summary in Figure 5A)

C57BL/6J male mice (own breeding facility) were single-caged, implanted i.p. with Minimitter transponders, housed at 30 °C, and fed HFD (the same as in Experiment 1) for 2 months. Subsequently, the mice (at age 20–21 weeks) were placed in the INCA chambers, and after 44 h (day 1) a 4-day treatment with s.c. injections of either 35 μg FGF21 or vehicle (see above; twice daily: 8 am and 6 pm) was started. On day 4, approximately 6 h after the last dose of FGF21/vehicle, the mice were euthanized and tissue samples collected as shown in Experiment 1.

**Figure 5:**
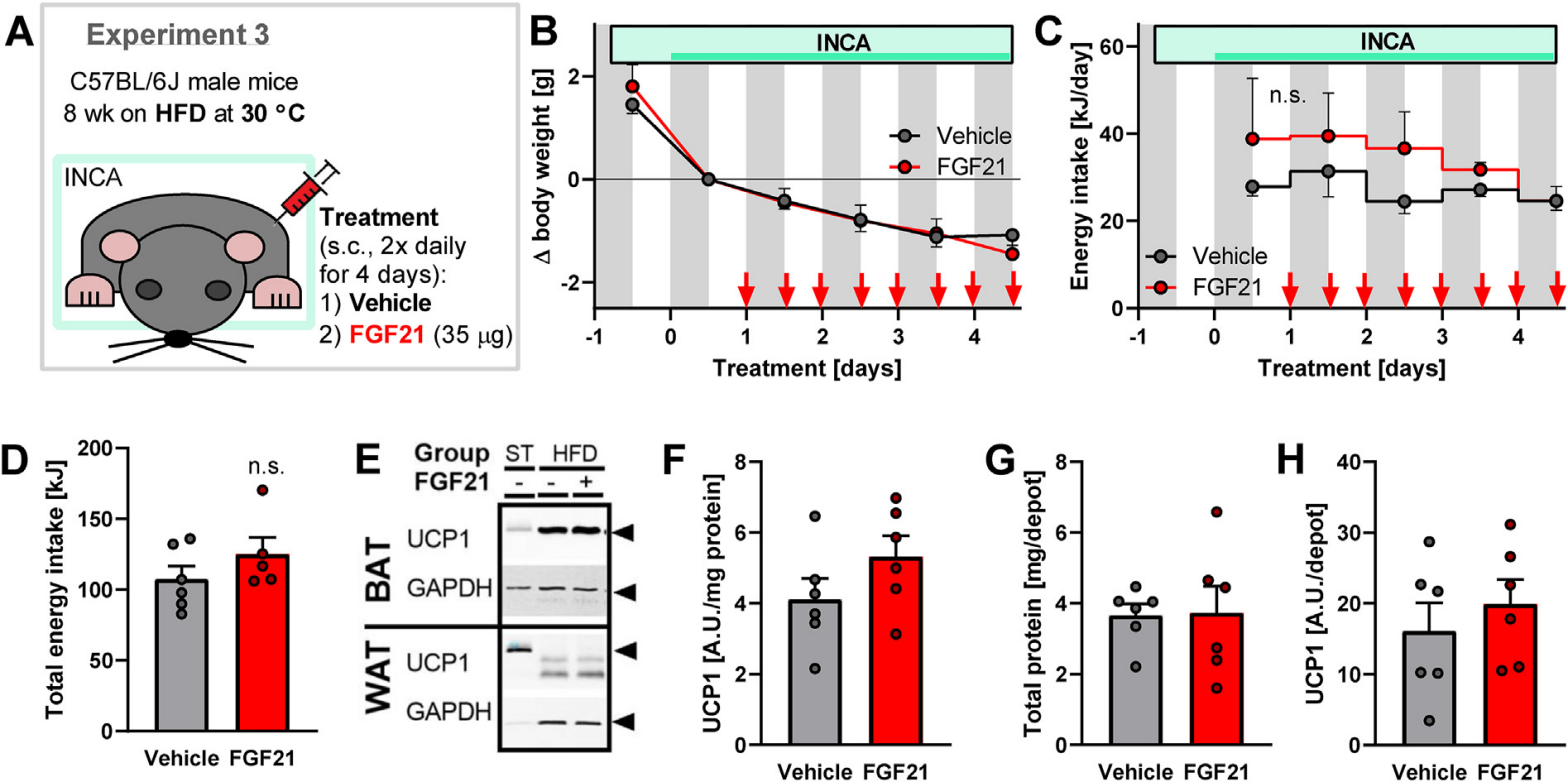
**Bodyweight, energy intake, and UCP1 content in FGF21-treated HFD-fed mice.** Experiment 3: C57BL/6J mice on HFD treated twice daily with 35 μg FGF21 or vehicle for 4 days (n = 6). The same mice as in Figure 6. [A] Summary of the experimental setup. See Methods for further details. [B] Development of body weight during the treatment. Grey rectangles represent the dark period of the day; red arrows represent the time of individual FGF21/vehicle injections; the light green box represents the time when mice were placed in the INCA chamber, and the darker green line represents the time of measurements showed in Figure 6. Body weight before the 1st injection (i.e. after a day of habituation to metabolic chamber) was set to 0. [C] Energy intake during the treatment. [D] Total energy intake during the 4 days of treatment. [E] Representative immunoblots showing UCP1 and GAPDH in BAT (2 μg protein loaded per well) and WAT (20 μg protein per well) of HFD-fed mice at thermoneutrality. For comparison, samples of HFD-fed mice housed at 20 °C (the same as in Figure 3E) were included. 2 μg of the same standard (ST) BAT sample were loaded on all the blots in this and other experiments and used for normalization, making the quantified UCP1 amount comparable among the experiments. Black arrows on the right show the sizes of UCP1 (33 kDa) and GAPDH (37 kDa). [F] Amount of UCP1 in interscapular BAT expressed per unit of protein (quantification of immunoblot band intensity). [G] Total protein amount in interscapular BAT. [H] Total UCP1 protein amount in whole interscapular BAT depot (calculated from UCP1 per protein in F multiplied by total protein in G). Statistics: Two-way repeated-measures ANOVA followed by Sidak's multiple comparison test for individual time points (B–C); Student's t-test (D, F–H) for comparison of the vehicle and FGF21-treated groups. No significant differences were found.

#### 2.1.4 Experiment 4 (see also a scheme in Figure 7A)

Male UCP1-deficient mice and their controls (UCP1^−/−^ and ^+/+^; C57BL/6J background, provided by Prof. Martin Klingenspor [14] but created by Prof. Leslie Kozak [15]) were bred and housed at 30 °C and fed chow (the same as in Experiment 1). At the age of 12–14 weeks, 3-day FGF21/vehicle treatment (as in Experiment 2) was started (day 1). During the first three injections (before the start of INCA measurements), tail surface temperature (app. 0.5 cm from tail root) was assessed by infrared camera FLIR E75 (FLIR): the temperature was always measured prior to the injection and 1 h after the injection). The change in tail temperature was calculated (tail temperature after the injection minus tail temperature before the injection). On day 2 (after the 3rd injection), mice were placed in the INCA chambers for 36 h. On the day of dissection (day 3), the mice received the last dose of vehicle/FGF21 and 1 h later a dose of CL 316,243 (as in Experiment 1). After 3–5 h, the mice were euthanized and samples collected as in Experiment 1.

**Figure 7:**
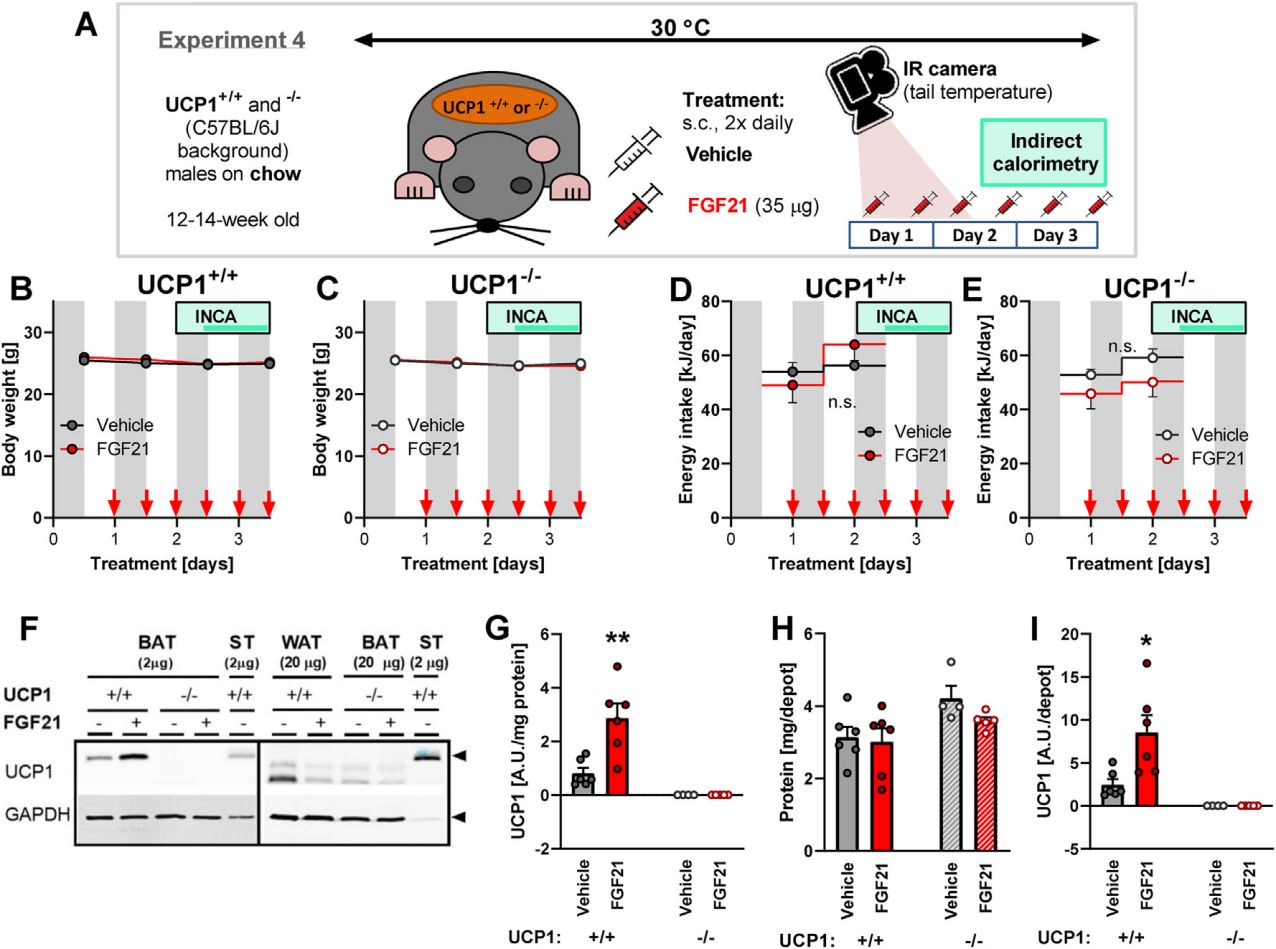
**FGF21 effects on body weight, energy intake, and UCP1 content in UCP1**^**+/+**^**and UCP1**^**−/−**^**mice.** Experiment 4: UCP1^+/+^ and UCP1^−/−^ mice (C57BL/6J background) on chow treated twice daily with 35 μg FGF21 or vehicle for 4 days (n = 6–7). Same mice as in part of Figure 8. [A] Scheme of the experimental setup. See Methods for further details. [B–C] Development of body weight of UCP1^+/+^ (B) and UCP1^−/−^ mice (C) during the treatment. Grey rectangles represent the dark period of the day; red arrows represent the time of individual FGF21/vehicle injections; light green box represents the time when mice were placed in the INCA chamber, and the darker green line represents the time of measurements shown in Figure 8A–F. [D-E] Energy intake of UCP1^+/+^ (D) and UCP1^−/−^ mice (E) during the treatment. [F] Representative immunoblots showing UCP1 and GAPDH in tissues of UCP1^+/+^ and UCP1^−/−^ mice at thermoneutrality. The same standard (ST) BAT sample was loaded on all the blots in this and other experiments and used for normalization, making the quantified UCP1 amount comparable among the experiments. WAT samples from UCP1^−/−^ were not collected; thus, 20 μg of BAT samples are used as a negative control in the right panel instead. Black arrows on the right show the sizes of UCP1 (33 kDa) and GAPDH (37 kDa). [G] Amount of UCP1 in interscapular BAT expressed per unit of protein (quantification of immunoblot band intensity). [H] Total protein amount in interscapular BAT. [I] Total UCP1 protein amount in the entire interscapular BAT depot (calculated from UCP1 per protein in G multiplied by total protein in H). Statistics: Mixed-effects model (REML) followed by Sidak's multiple comparison test for individual time points (B-E; no significant differences between treatments were found); Student's t-test (G–I). Significant difference between treatments is marked with ∗. ∗ represents p < 0.05, ∗∗p < 0.01.

### 2.2 Indirect calorimetry (INCA)

The INCA system (Somedic) was used to assess O_2_ consumption (V_O2_) and CO_2_ production (V_CO2_; 2 min resolution). The incorporated telemetry system (Respironics) provided data on both body temperature and physical activity. These parameters were measured either at a constant ambient temperature of 30 °C (Experiments 2–4) or at ambient temperatures ranging between 14 °C and 32 °C (Experiment 1). Prior to the measurement itself, the mice were habituated to the new environment in the INCA chambers at least overnight. O_2_ consumption and body temperature are presented as running mean curves (each point is a running mean of 5 values from 10 min periods to minimize the potential effect of individual outlying values) and also as means or sums of whole light and dark periods.

Energy expenditure was calculated according to the formula: Energy expenditure [W] = 0.272 V_O2_ [ml/min] + 0.0767 V_CO2_ [ml/min] [16]. In experiment 1, mice were exposed to various temperatures (3 h at each temperature, see Figure 1A); minimal stable energy expenditure was calculated from mean V_O2_ and V_CO2_ obtained during a consecutive 20 min period at the lowest stable V_O2_ values (usually by the end of each temperature period). The values of body temperature from the same 20 min were used for the calculation of mean basal body temperature at each ambient temperature. The sum of physical activity was measured for the entire exposure time at the respective temperature, except for the first 30 min habituation (i.e. for 2.5 h, then recalculated as A.U./h)

Thermal conductance was calculated as given in [17] using the following formula:

Thermal conductance [W/°C] = energy expenditure [W] / (body temperature minus ambient temperature [°C]

### 2.3 Western blot

Protein extracts were prepared from frozen samples of adipose tissues as described previously [18]. Briefly BAT (approximately tissue:buffer 1:9) and inguinal WAT (tissue:buffer 1:4) were homogenized in ice-cold lysis buffer (50 mM Tris, pH 7.4; 1 mM EDTA; 0.15 M NaCl; 1 mM benzamidine; 1 mM dithiothreitol; 50 mM sodium fluoride; 5 mM pyrophosphate tetrasodium; 1 mM phenylmethylsulfonylfluoride; 0.2% Triton X-100; 1% glycerol; 10 mg/ml aprotinin; 10 mg/ml pepstatin; 10 mg/ml leupeptin and Phosphatase Inhibitor-Mix I (Serva, Heidelberg, Germany) using a ball mill MM40 (Retsch, Germany; 30 s-1, 3 min) and then centrifuged at 4 °C (2000 g, 10 min). The aqueous interphase between the pellet of debris and the fatty upper layer was aliquoted and frozen. The protein concentration was assessed by the bicinchoninic acid method, and protein amount in whole tissue was calculated.

BAT (2 μg protein/well) and WAT (20 μg protein/well) samples were loaded on 10% Tricine SDS-PAGE gel. Electrophoresis and western blotting were performed as described earlier [19]. In some experiments, additional controls were included: BAT samples of UCP1-deficient (as a negative control of UCP1 content), or BAT and WAT samples of HFD-fed mice treated as the mice in Experiment 1 but housed at 20 °C (as an example of partially recruited UCP1 capacity). UCP1 was detected using Human/Mouse UCP1 Antibody (Monoclonal Mouse IgG2B Clone # 536,435, RD Systems, Catalogue Number: MAB6158, 1:1000) and secondary Goat anti-Mouse IgG (H + L) Cross-Adsorbed Secondary Antibody (A-21057, Alexa Fluor 680). Equal protein loading was checked by evaluation of the amount of GAPDH protein, using GAPDH (14C10) Rabbit mAb (#2118, Cell Signaling, 1:1000) and IRDye® 800CW Donkey anti-Rabbit IgG Secondary Antibody (Li-cor). Membranes were scanned using an Odyssey ®Infrared imaging system (Li-cor) and visible protein bands were quantified using Image Lab software (Bio-Rad Laboratories). Subsequently, UCP1 amount per 1 μg total protein or whole adipose tissue depot was recalculated and expressed (i.e. the amount of UCP1 per unit of protein was multiplied by total protein amount in the depot [20]). Importantly, all band intensities were normalized to the same standard sample loaded to each gel, so the A.U. is comparable between the experiments.

## 3 Results

To allow for discrimination of the effects of FGF21 on energy expenditure and body temperature control, we have examined here, in several models, the relationship between these parameters and related this to the recruitment, activation, and necessity of UCP1 for the observed effects of FGF21.

### 3.1 FGF21 raises defended body temperature

If the increase in body temperature caused by FGF21 is secondary to an imbalance between heat production and heat loss, the magnitude of the increase should be altered when the ambient temperature is altered. Particularly, the higher body temperature observed in FGF21-treated mice should be normalized if the ambient temperature is reduced and heat loss is thus facilitated. If such normalization of body temperature in the cold were *not* to take place, the increased body temperature would not be hyperthermia but would instead reflect an alteration in body temperature control. Thus, to establish the nature of the effect of FGF21 on body temperature, we assessed the body temperature of control and FGF21-treated mice habituated to thermoneutrality and acutely exposed to a series of ambient temperatures.

For this, mice on a normal chow diet and acclimated to thermoneutrality were implanted with osmotic minipumps delivering either 30 μg/d FGF21 or its vehicle, and with Minimitters to follow body temperature (Figure 1A). All mice (including the control ones) lost some weight over the following week (Figure 1B “chow”; Figure 1C). FGF21-treated mice showed an additional small but clear body weight decrease (Figure 1C), in the absence of any significant negative change in food intake (Figure 1E).

**Figure 2:**
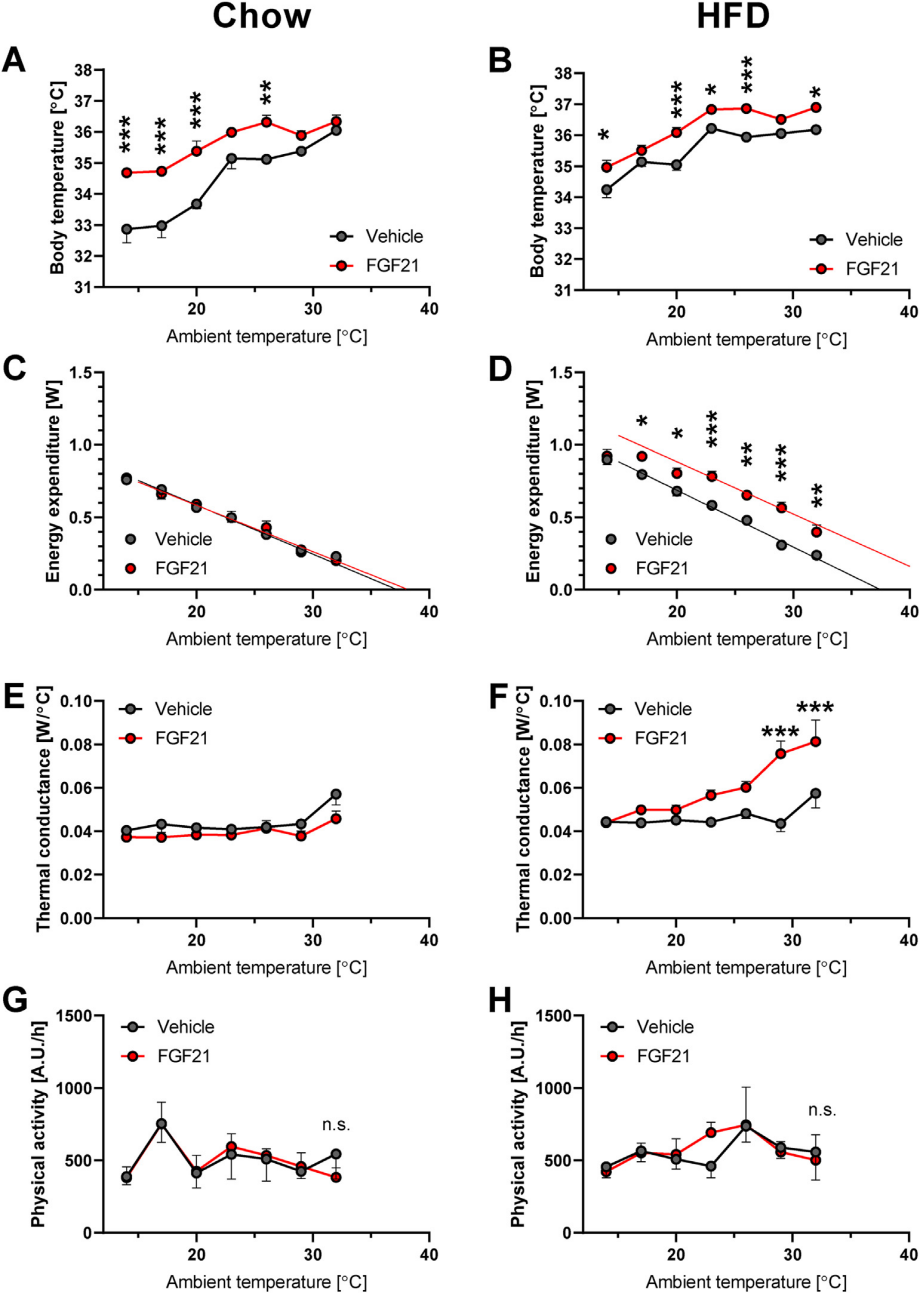
**Effects of FGF21 on body temperature and energy expenditure at various ambient temperatures.** Experiment 1: The same mice as in Figure 1 and part of Figure 3. [A-B] Body temperature as a function of ambient temperature in chow-fed (A) and HFD-fed mice (B). [C-D] Energy expenditure as a function of ambient temperature in chow-fed (C) and HFD-fed mice (D): lines represent extrapolated linear regression of values in the range of ambient temperatures 23–29 °C (the body temperature is stable in this range). Note that points representing vehicle- and FGF21-treated groups may overlap in this and similar graphs. [E-F] Thermal conductance (calculated as energy expenditure divided by the difference between ambient and body temperature) as a function of ambient temperatures in chow-fed (E) and HFD-fed mice (F). [G-H] Physical activity as a function of ambient temperature in chow-fed (G) and HFD-fed mice (H): each point represents a sum of 2.5-h measurement (i.e. entire period of exposure to the temperature except for the starting 30 min habituation). Statistics: Two-way repeated measures ANOVA (A, C, E, G) or mixed-effects model (REML, as some individual values are missing for technical reasons; B, D, F, H) followed by Sidak's multiple comparison test for individual ambient temperatures (if Student's t-test is used to compare the groups at each temperature point, the difference in body temperature is highly significant even at 29 °C (p < 0.02 in 2A and p < 0.003 in 2B); simple linear regression (C, D). Significant difference between FGF21 and vehicle treatments at each ambient temperature: ∗p < 0.05, ∗∗p < 0.01, ∗∗∗p < 0.001.

To examine the nature of the effect of FGF21 treatment on body temperature control, the mice were exposed to a range of temperatures, during which energy expenditure and body temperature were recorded. When exposed to ambient temperatures of 23–30 °C, the control mice maintained a body temperature of 35 °C (Figure 2A). When the ambient temperature was decreased below 23 °C, the body temperature of the control mice started to decline (black line in Figure 2A), principally as expected in those mice that were acclimated to thermoneutrality, and thus had limited thermogenic capacity [14].

Similar to the finding by Coskun [5], we found that at 23 °C (i.e. approximately as in [5]), the FGF21-treated mice had a higher body temperature than control mice (Figure 2A; see Statistics section of Figure legend). If this difference is hyperthermia, i.e. a consequence of the production of more heat than can be fully dissipated, the difference between the FGF21-treated and the control mice would be reduced as the environmental temperature is decreased, since the extra heat would be more easily dissipated in a colder environment. However, this was not the case. The body temperature remained elevated by the impact of FGF21 over a broad range of ambient temperatures (Figure 2A); the effect even seemed to be more pronounced at lower ambient temperatures. This clearly indicates that the higher body temperature is due to a shift in body temperature set point, not by an induction of thermogenesis.

This conclusion is considerably reinforced when the rate of heat production (energy expenditure) is followed in these mice (Figure 2C). In the control mice, exposure to lower ambient temperatures resulted, as expected, in an increase in energy expenditure (thermogenesis) directly in proportion to the lowering of the temperature [21]. Remarkably, despite the higher body temperature induced by FGF21 (Figure 2A), the FGF21-treated mice did not demonstrate a higher energy expenditure than the control mice at any ambient temperature (Figure 2C). The higher body temperature was thus clearly not secondary to increased heat production, rather it occurred independently.

Two important conclusions can be drawn from these experiments: FGF21 can increase body temperature independently of heat production – and FGF21 does not obligatorily increase energy expenditure. Additionally, the result demonstrates that a change in body temperature cannot unreservedly be interpreted as an indication of an alteration in energy expenditure.

### 3.2 FGF21 raises defended body temperature also in diet-induced obese mice

Due to the interest in FGF21 as an anti-obesity substance, we also examined the effect of FGF21 on body temperature control in mice that had been HFD-fed (at thermoneutrality) and were thus obese (≈50 g versus chow-fed ≈30 g body weight) (Figure 1B). Also in these non-FGF21-treated mice, the surgery in itself resulted in a decrease in body weight (Figure 1D), clearly potentiated by a marked reduction of food intake (Figure 1F). FGF21 treatment amplified the reduction of body weight (Figure 1D) without further affecting food intake (Figure 1F), but the effect of FGF21 per se on body weight was not different from that in chow-fed mice (both about 3 g, compare the difference between vehicle- and FGF21-treated groups in Figure 1C,D).

Similar to the case in chow-fed mice, HFD-fed mice maintained a stable body temperature at ambient temperatures above 23 °C but showed somewhat lower body temperatures when the ambient temperature was decreased below 23 °C (Figure 2B). In a very systematic way, the body temperature of the FGF21-treated mice was consistently about 1 °C higher than that of the controls (Figure 2B), i.e. the difference in body temperatures was present at thermoneutrality and did not diminish in the cold – despite an environment that would allow more heat dissipation. Therefore, there was no indication that the increased body temperature was hyperthermia, rather it must again reflect an alteration in body temperature control.

Also in the HFD-fed mice, energy expenditure increased linearly with decreasing ambient temperature (Figure 2D), as expected, and there was no indication that the obesity provided any insulation against the cold (in agreement with earlier observations [22]) (i.e. the slope was not shallower in the HFD-fed mice).

In clear contrast to the case in the chow-fed mice, in the HFD-fed mice, FGF21 induced increased energy expenditure at all tested temperatures (except the lowest (Figure 2D). Notably, the increase was the same at all ambient temperatures (at least in the range of 23–33 °C), i.e. it was not due to an alteration in insulation (the slope was the same as in the control mice). The increase in energy expenditure thus remained even in the thermoneutrality zone (≈30 °C) and the slope extrapolated to a higher defended body temperature than in the chow-fed mice. Thus, FGF21 *can* induce an increase in energy expenditure but this is dependent on experimental conditions, such as diet.

We further calculated thermal conductance, i.e. the measure of the ease with which heat is dissipated to the environment (formally, conductance mathematically equals 1/insulation – an increase in conductance reflects physiological attempts to dissipate extra heat) [17,23]. The above data show that FGF21 had only a marginal effect on thermal conductance in chow-fed mice (Figure 2E). In FGF21-treated HFD-fed mice, thermal conductance showed a remarkable increase with increasing temperature (Figure 2F). The change in conductance implies that the mice were (successfully) fighting *against* the FGF21-induced increase in energy expenditure in order to maintain a constant body temperature, as heat dissipation becomes more difficult at higher ambient temperatures. The increase in heat production would therefore not seem to be recruited as a means to increase body temperature but rather as an independent phenomenon.

Although energy expenditure could be affected by physical activity, there was no difference in physical activity between vehicle and FGF21-treated mice on either diet or at any temperature (Figure 2G,H).

### 3.3 FGF21 chronically activates β_3_-adrenergic thermogenesis in HFD-fed mice

No increase in energy expenditure by FGF21 was seen in chow-fed mice but was evident in HFD-fed mice. To investigate the origin of this extra energy expenditure, we examined whether adrenergic thermogenesis was induced, particularly β_3_-adrenergic thermogenesis. We returned the mice to thermoneutrality (32 °C) and injected them (unanaesthetized) with the β_3_-adrenergic agonist CL 316,243. In unanaesthetized mice, the response to such an injection consists of two phases: an initial but transient stress response (≈30 min) and a longer time (≈8 h) thermogenic response.

Prior to the CL 316,243 injection, the body temperature of chow-fed mice was again somewhat higher in the FGF21-treated than in the control group (Figure 3A). Again, basal values of energy expenditure did not differ between control and FGF21-treated animals on chow diet (Figure 3B), in agreement with the absence of effect of FGF21 seen before (Figure 2C). Activation of UCP1 by the β_3_-adrenergic agonist resulted in a robust rise both in body temperature (Figure 3A) and energy expenditure (Figure 3B). Thus, in these chow-fed mice, there was no effect of the FGF21 treatment on the response to CL 316,243.

In the HFD-fed mice, the outcome was markedly different. The robust basal difference in body temperature between control and FGF21-treated mice was eliminated after CL 316,243 injection (Figure 3C), as was the difference in energy expenditure (Figure 3D). Indeed, in these mice, it would appear at first sight that CL 316,243 had lost its thermogenic effect in the FGF21-treated mice. However, an alternative, but more probable, interpretation is that the β_3_-adrenergic thermogenesis was already fully activated (due to the FGF21 treatment) before the CL 316,243 injection. There is thus an apparent absence of effect of CL 316,243 – though it may result from the thermogenic capacity already being fully stimulated.

To investigate whether the effects of FGF21 are mediated fully by the central nervous system and sympathetic nerves, we performed an experiment in HFD-fed mice (Figure 3E) in which we blocked adrenergic signaling by the general β blocker propranolol (Figure 3F). In this experiment, we delivered FGF21 by twice-daily s.c. injection to avoid the post-surgery stress after osmotic pump implantation. The basal body temperature (Figure 3G) and energy expenditure (Figure 3H) of control mice and mice after 8 injections of FGF21 were comparable to those in the previous experiment, in which osmotic pumps were used (Figure 3C–D).

Propranolol is a non-selective β-blocker with a high affinity for β_1_ and β_2_ receptors and lower affinity for β_3_ receptors [24], easily crossing the blood-brain barrier. Besides its well-known effects on the cardiovascular system, it also affects brain circuits: It counteracts centrally elicited fever [25].

Propranolol did not affect body temperature (Figure 3G) or energy expenditure (Figure 3H) in control mice. In contrast, the elevated body temperature of FGF21-treated animals was decreased by propranolol down to the levels of control animals (Figure 3G). Similarly, the energy expenditure of the FGF21-treated mice was decreased after the propranolol injection (Figure 3H). This indicates that elevated body temperature and energy expenditure were induced via the β-adrenergic effect. The subsequent dose of CL 316,243 increased body temperature and energy expenditure to similar levels as in the previous experiment (compare Figure 3G,H to Figure 3C,D). Moreover, in the animals that received saline before CL 316,243, the subsequent additional injection of propranolol did not decrease energy expenditure and body temperature. The specific β_3_-adrenergic agonist CL 316,243 could therefore fully overcome the effect of the rather weak β_3_-antagonist propranolol.

In conclusion, most of the effects of FGF21 on body temperature and energy expenditure were mediated by adrenergic pathways (e.g., central nervous circuits and the sympathetic nervous system).

### 3.4 FGF21 increases UCP1 amounts in BAT, but not in WAT

The thermogenic effect of FGF21 would be expected to be mediated via activation and possibly recruitment of UCP1 in BAT and possibly in WAT. Studies have suggested that induction of UCP1 gene expression in WAT (i.e., browning) is the crucial process for the thermogenesis [7], as induction of UCP1 gene expression by FGF21 was observed in WAT, though reportedly only to a smaller or no extent in BAT [5,7].

To test this hypothesis, we analysed the content of UCP1 in interscapular BAT (the largest BAT depot in mice) and inguinal WAT (the largest WAT depot prone to browning) in the mice from the first experiment (Experiment 1). The UCP1 protein levels (Figure 3I), rather than the UCP1 mRNA levels assessed in earlier reports, closely correlates with the capacity for non-shivering thermogenesis [26]. We, therefore, quantified UCP1 protein content per unit of protein in BAT (Figure 3J left). UCP1 content per mg protein was higher in BAT of HFD-fed than chow-fed mice, in agreement with earlier observations [[27], [28], [29], [30], [31], [32]]. On either diet, FGF21 resulted in a robust increase in UCP1 levels per mg protein, in HFD-fed mice almost reaching the levels seen in mice housed at 20 °C (Figure 3J left). The total protein content in the depot was similar in all experimental groups (Figure 3J middle). Thus, the total amount of UCP1 protein in BAT was increased by FGF21 both in chow- and HFD-fed mice (Figure 3J right). This would imply a marked increase in total thermogenic capacity in the FGF21-treated mice. Nonetheless, as the total UCP1 amount in BAT of the FGF21-treated chow-fed mice reached only the level found in untreated HFD-mice and half of that seen in FGF21-treated HFD-fed mice, the induction in chow-fed mice may be insufficient to mediate a significant effect on energy expenditure.

When assessing UCP1 protein in inguinal WAT, we could not detect any UCP1 protein (although we loaded 10x more WAT protein than BAT protein) (Figure 3I), as in [6].

Thus, in contrast to earlier suggestions implying a recruiting effect of FGF21 in WAT but not in BAT [7], we observed significant FGF21-induced recruitment only in BAT.

### 3.5 FGF21 does not induce pyrexia due to an immune response

**Figure 4:**
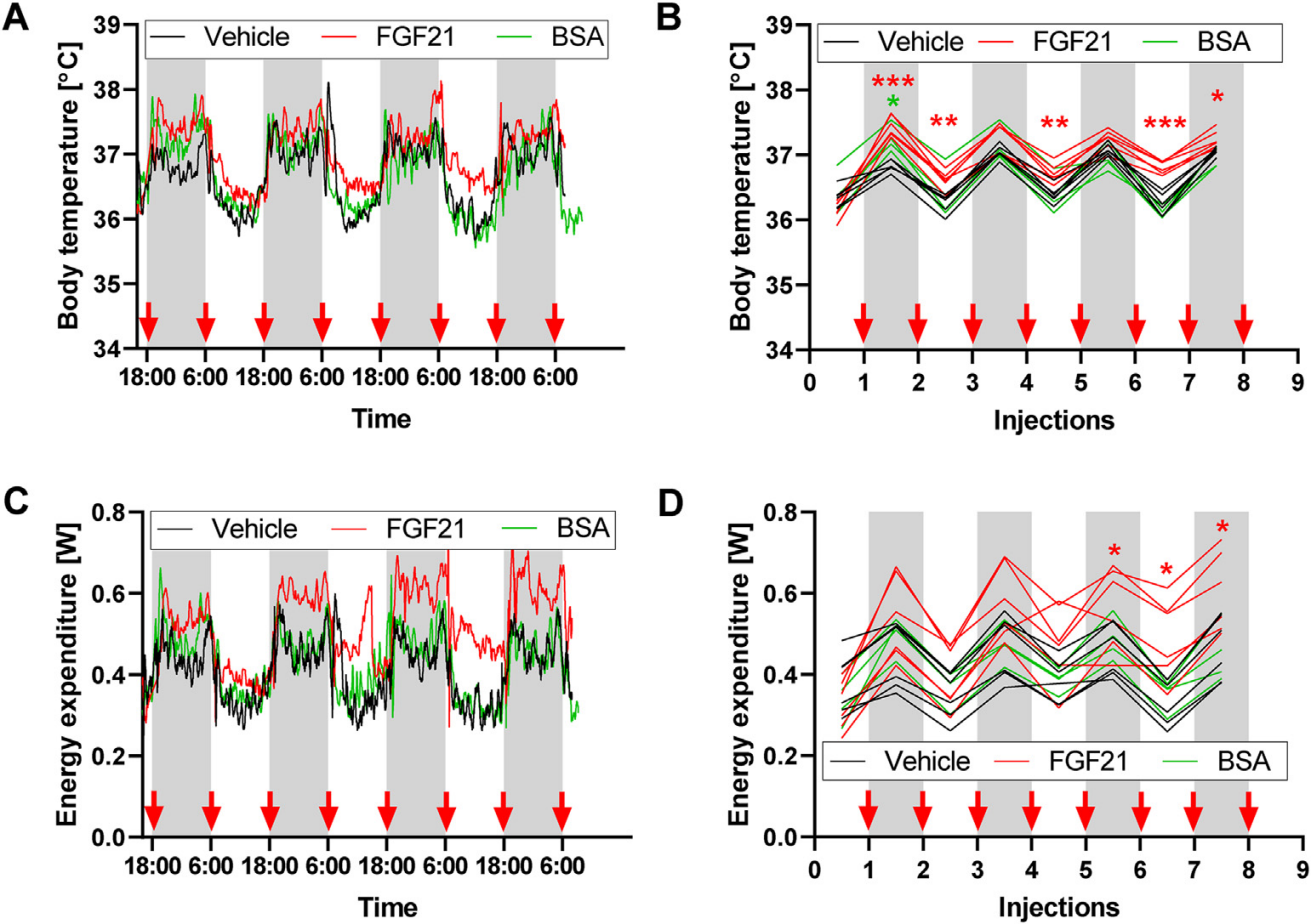
**Comparison of effects of FGF21 vs. albumin.** Experiment 2: The same mice as in parts of Figure 3, Figure 8. [A-B] Body temperatures at 30 °C during 4-day FGF21/vehicle/albumin (BSA) treatment: Average curves for individual groups (A); 12-h mean values of the individual mice for dark and light periods of each day (B). In these and similar graphs, grey rectangles represent dark periods of days, and red arrows indicate the times of individual FGF21/vehicle/albumin injections. [C-D] Body temperatures at 30 °C during 4-day FGF21/vehicle/BSA treatment: Average curves for individual groups (C); 12-h mean values of the individual mice for dark and light periods of each day (D). Statistics: 2-way repeated measures ANOVA and Dunnett's multiple comparison test for comparison of vehicle vs. FGF21 and vs. albumin in the individual time points (B, D). ∗ represents p < 0.05, ∗∗p < 0.01, ∗∗∗p < 0.001.

Similar to the case in most previous studies of FGF21, the mice in this study were given recombinant *human* FGF21 protein. Although we only studied relatively short-term treatment and immune reactions to human FGF21 in mice have not been reported, we were cautious about the potential interference of a specific immune reaction to the human protein, given the observed pyrexic effects of FGF21. To examine the hypothesis that FGF21 raises body temperature via its specific receptors rather than through induction of an immune response, we compared vehicle- and FGF21-treatment with treatment by *bovine* serum albumin (in the same concentration as FGF21; Figure 4). Human FGF21 vs murine FGF21 have similar levels of homology as do murine albumin and bovine albumin (FGF21: 75% identity and 80% similarity; albumin: 70% identity and 84% similarity), so the probability of immunogenic effects should be similar.

FGF21 (in comparison to vehicle) again robustly increased body temperature from the start of the treatment (Figure 4A,B) and also affected energy expenditure, although this effect did not reach statistical significance before the 5th injection, mainly due to a higher variance among energy expenditure data (Figure 4C,D). In contrast, the effect of bovine albumin on body temperature (Figure 4A,B) and energy expenditure (Figure 4C,D) did not differ from the effect of vehicle (except for body temperature in the night after the 1st albumin injection – Figure 4B).

Thus, the observed pyrexic effect of human FGF21 in mouse is unlikely to be caused by an immune reaction to this protein.

### 3.6 Temporal disconnection between FGF21 effects on body temperature and energy expenditure

In order to elucidate the connection between increases in body temperature and energy expenditure (i.e. whether either of these parameters precedes the other), we studied the time frame of the FGF21 effects. As an increase in energy expenditure by FGF21 was only observed in HFD-fed mice, the time effect was followed in these mice (see experimental summary in Figure 5A).

For these experiments, thermoneutrally housed, diet-induced obese mice, placed constantly in the chambers for INCA and fitted with Minimitter probes, were treated with s.c. injections of FGF21 twice daily for 4 days.

**Figure 6:**
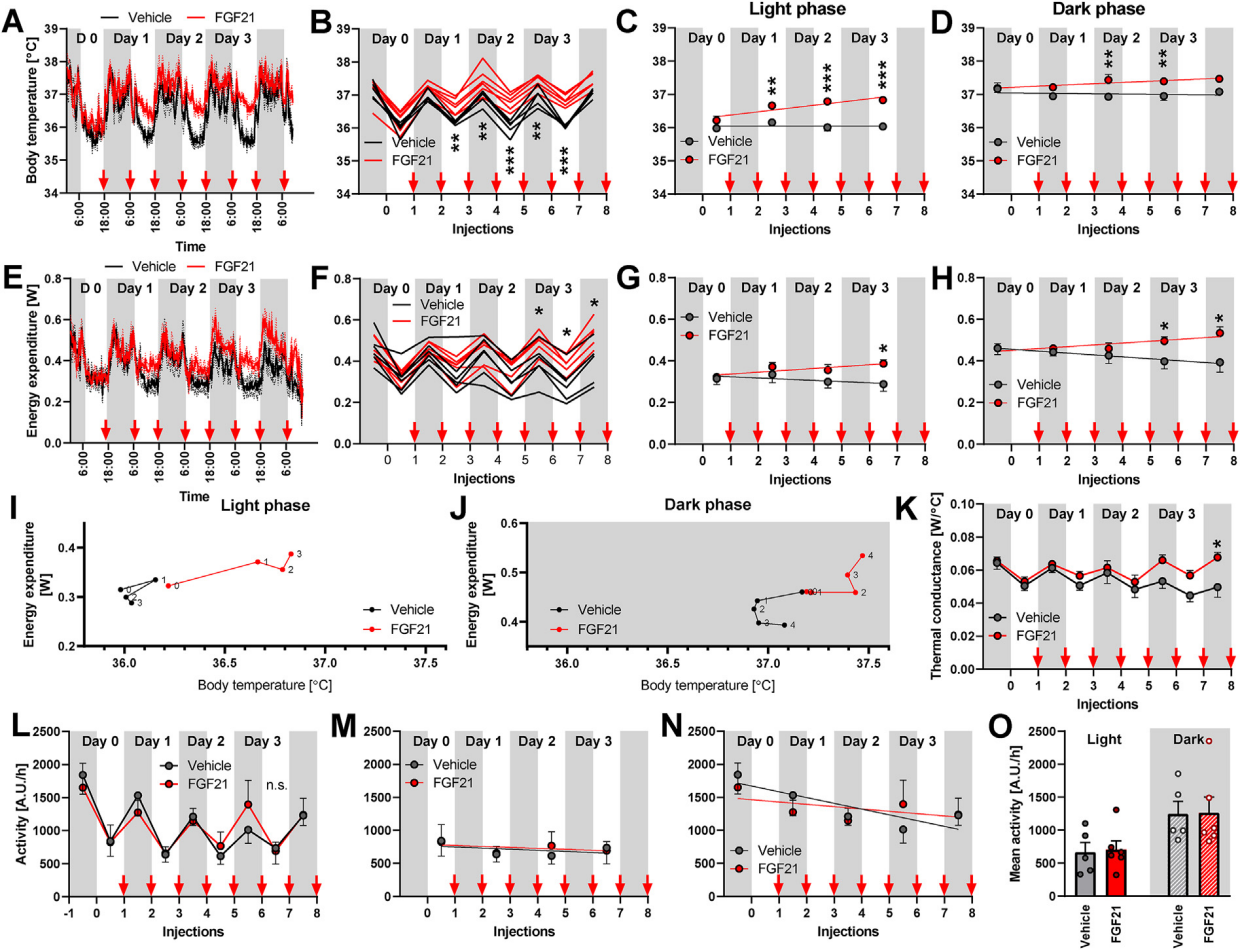
**Development of FGF21-induced changes over time.** Experiment 3: The same mice as in Figure 5. [A-D] Body temperatures at 30 °C during 4-day FGF21/vehicle treatment: Average curves for individual groups (A); 12-h mean values of the individual mice for dark and light periods of each day (B); changes during the light (C) and dark phase of the day (D). FGF21 slope in C is significantly non-zero (p = 0.003) and both slopes are significantly unequal (p = 0.005), while the slopes in D are neither significantly non-zero nor unequal. In these and similar graphs, grey rectangles represent dark periods of days, and red arrows indicate the times of individual FGF21/vehicle injections. [E-H] Energy expenditure at 30 °C during the 4-day FGF21/vehicle treatment: Average curves for individual groups (E) and 10-h mean values of the individual mice for dark and light periods of each day (F; data obtained 60 min before and after the light switch were omitted for this calculation); changes during the light (G) and dark phase of the day (H). FGF21 slope in H is significantly non-zero (p = 0.02) and both slopes are significantly unequal (p = 0.007), while the slopes in G are neither significantly non-zero, nor unequal. [I-J] Energy expenditure as a function of body temperature during the treatment (shown separately for the light [I] and dark [J] period of the day. Each point represents the average of all mice on the given treatment day and is labelled by the day number. Note the different y axis in I vs. J. [K] Development of thermal conductance (calculated as energy expenditure divided by the difference between ambient and body temperature) during the treatment. [L-O] Physical activity at 30 °C during 4-day FGF21/vehicle treatment; mean 12-h physical activity during the treatment (L); changes during the light (M) and dark phase of the day (N); mean day time and night time physical activity during the whole treatment (O). The slopes in M are neither significantly non-zero, nor unequal. Vehicle slope in N is significantly non-zero (p = 0.02) but the slopes are not significantly unequal. Statistics: Two-way repeated measures ANOVA (F, K) or mixed-effects model (REML; B, M) followed by Sidak's multiple comparison test for individual time periods (B, F, K, L); linear regression testing difference of the slopes and their departure from linearity (C, D, G, H, M, N; for the outcome see the figure legend above); Student's t-test for the difference between vehicle- and FGF21-treated group (O). A significant difference between treatments is marked with ∗. ∗ represents p < 0.05, ∗∗p < 0.01, ∗∗∗p < 0.001.

Despite the effort to minimize any stress, the transfer of mice to the INCA chamber resulted in an initial drop in body weight (Figure 5B, before the 1st injection). This presumed stress was probably also reflected by a high initial physical activity level during the active period (nighttime) (Figure 6N), which slowly decreased during the entire experiment (Figure 6L and N). The total physical activity was not affected by the FGF21 treatment (Figure 6O). During this (rather short) 4-day treatment, FGF21 did not have any significant effect on body weight and food intake (Figure 5B–D).

Body temperature and energy expenditure exhibited strong circadian rhythmicity, as expected. FGF21 treatment significantly increased body temperature from the start of the treatment (Figure 6A,B), and the magnitude of the effect further increased during the following days. The body temperature increase was especially pronounced during the daytime (Figure 6C) and less significant at night-time (Figure 6D). The effect of FGF21 on energy expenditure followed a similar pattern (Figure 6E,F), but as there was considerably larger variation among the data, the effect did not reach statistical significance until the 5th injection (Figure 6F). In contrast to body temperature, the gradual rise in energy expenditure was manifest to a similar extent during daytime (Figure 6G) and at night-time (Figure 6H). This difference again stresses the distinction between the effect of FGF21 on body temperature and on energy expenditure and implies that they are distinct entities.

To analyse the relationship between the changes in body temperature and energy expenditure in the FGF21-treated mice, we plotted energy expenditure on each day as a function of body temperature on that day (Figure 6I,J). During the daytime, body temperature increased rapidly, accompanied by small changes in energy expenditure (Figure 6I). At night-time, FGF21 seemed to increase body temperature initially (Figure 6J, between days 1 and 2) and energy expenditure then successively increased during the following days, without corresponding body temperature changes. Thus, again, the body temperature and energy expenditure were not functionally correlated. Similar to the case in the previous experiment (Figure 2F, at 30 °C), thermal conductance was elevated by FGF21 (Figure 6K).

### 3.7 Increases in body temperature and energy expenditure by FGF21 treatment are not dependent on the induction of significant changes in UCP1 amount

In the HFD-fed mice in Experiment 1, the FGF21-induced increase in energy expenditure correlated with a higher content of UCP1 (Figure 3D,J), suggesting that an increase in UCP1 protein levels may be important for the effect of FGF21 on energy expenditure. Thus, we assessed the UCP1 content in the present experiment as well. The UCP1 content in BAT tended to be elevated in the FGF21-treated group by the end of this short-term FGF21 treatment, but this increase did not reach significance (Figure 5F–H). Thus, the enhancement of energy expenditure and body temperature occurred before there was any robust increase in UCP1 and was therefore independent of an increase in UCP1. We confirmed the absence of detectable UCP1 protein amounts in the inguinal WAT (Figure 5E).

The total amount of UCP1 represents the capacity of the animal for non-shivering thermogenesis but does not need to correlate with the actual energy expenditure, since UCP1 may or may not be activated. Although we could observe both a significant increase in UCP1 without a corresponding increase in energy expenditure (as in the chow-fed mice in Experiment 1) and an increase in energy expenditure without a corresponding increase in UCP1 (as in Experiment 2), this finding does not exclude a role of UCP1 in FGF21-induced energy expenditure. Also, an FGF21-driven increase in the activity of pre-existing UCP1 may have played a role in the increase in energy expenditure in that experiment. The only way to specifically investigate the role of UCP1 in the effects of FGF21 treatment is to use a UCP1-deficient model.

### 3.8 UCP1 mediates the effect of FGF21 on energy expenditure, not on body temperature

**Figure 8:**
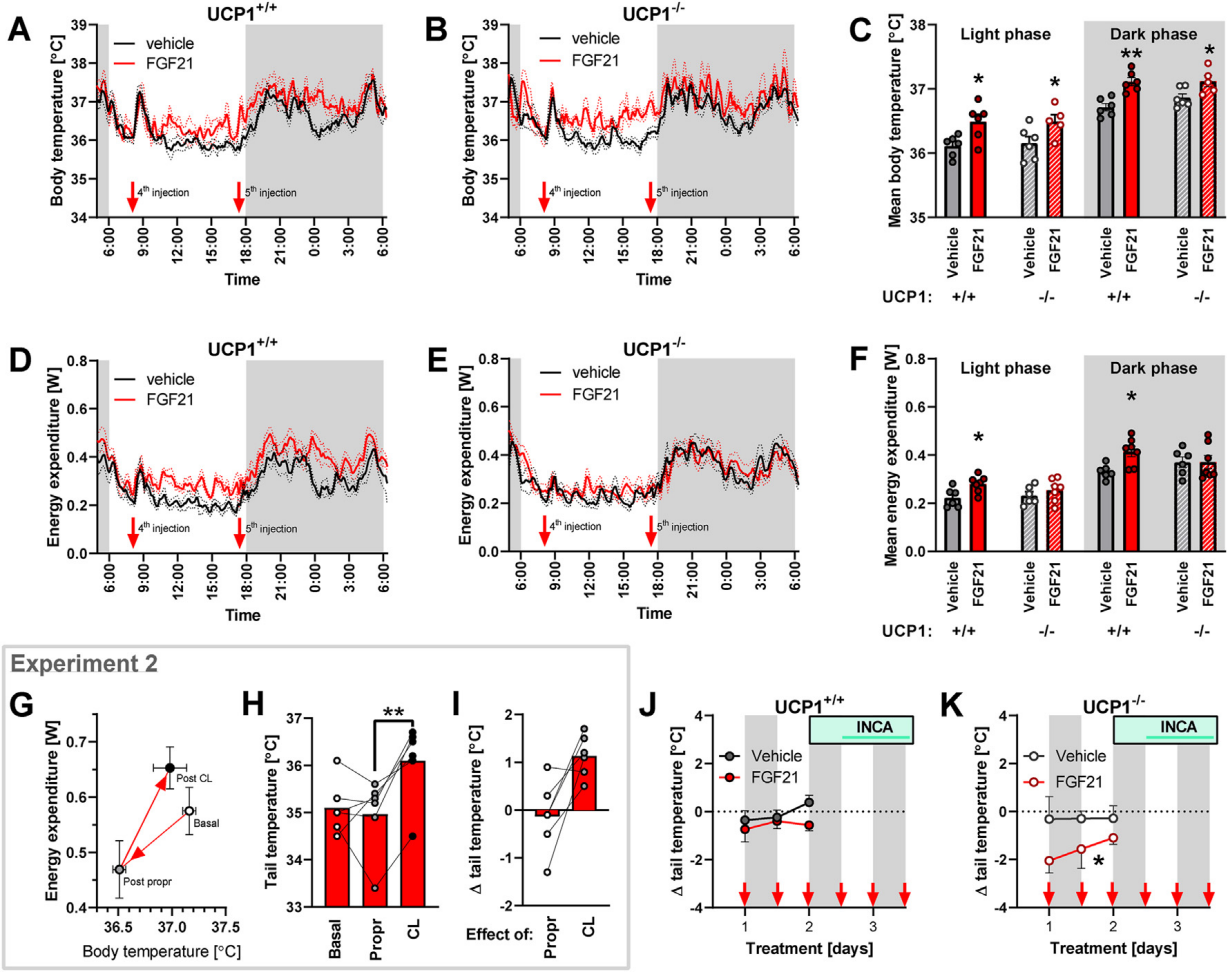
**FGF21 effects on body temperature and energy expenditure in UCP1**^**+/+**^**and**^**−/−**^**mice.** Experiment 4: Same mice as in Figure 7. Experiment 2: Same mice as in Figure 4 and part of Figure 3. [A-C] Body temperature at 30 °C during the 3rd day of FGF21/vehicle treatment: Mean curves for UCP1^+/+^ (A) and UCP1^−/−^ mice (B) and average body temperature during the light and the dark period of the day in both genotypes (C). [D-F] Energy expenditure at 30 °C during the 3rd day of FGF21/vehicle treatment: Mean curves for UCP1^+/+^ (D) and UCP1^−/−^ mice (E) and average energy expenditure during the light and the dark period of the day in both genotypes (F). [G] Changes in body temperature and energy expenditure after injection of propranolol and CL 316,243 (data corresponding to Figure 3G–H). [H] Tail temperature immediately before propranolol injection, 1 h later (and immediately before CL 316,243 injection), and 1 h after CL 316,243 injection (corresponding data to G). [I] Change in the tail surface temperature as an effect of injection of propranolol and CL 316,243 (calculated as difference between temperature before and after the respective injection – see H). [J-K] Change in the tail surface temperature 1 h after FGF21/vehicle injection in UCP1^+/+^ (G) and UCP1^−/−^ mice (H). According to 2-way repeated-measures ANOVA, there is a significant effect of time (p = 0.049) and interaction between time and treatment (p = 0.002) in UCP1^+/+^ (G) and a significant effect of treatment (p = 0.047) in UCP1^−/−^ (H). Statistics: Student's t-test (C, F); one-way repeated measures ANOVA followed by Tukey's multiple comparison test (H); two-way repeated-measures ANOVA followed by Sidak's multiple comparison test (J–K). A significant difference between treatments is marked with ∗. ∗ represents p < 0.05, ∗∗p < 0.01.

Previous literature is not unanimous on the question of the importance of UCP1 for the effects of FGF21 on energy expenditure. In one study, an FGF21-induced increase in energy expenditure was demonstrated at thermoneutrality even in UCP1-deficient mice [6]; in another, in UCP1-deficient mice (housed at sub-thermoneutral temperature), FGF21-induced increases in energy expenditure were attenuated [11]. Neither of the published studies examined whether UCP1 is necessary for FGF21-induced changes in body temperature. Therefore, to resolve the role of UCP1 in FGF21 effects on body temperature and energy expenditure, we applied a 4-day FGF21 treatment (s.c. injections twice daily, similar to the above) to chow-fed UCP1-deficient and control mice (referred to as UCP1^−/−^ and ^+/+^, respectively) (Figure 7A). This short treatment did not significantly affect body weight (Figure 7B,C) or food intake (Figure 7D,E). As in the two previous experiments, no UCP1 protein was detected in the inguinal WAT of UCP1^+/+^ mice (Figure 7F), though FGF21 increased the content of UCP1 in the BAT of these mice (Figure 7G,H, and I). Body temperature (Figure 8A–C) and energy expenditure (Figure 8D–F) were assessed between fourth and sixth FGF21/vehicle injections. As expected, the circadian rhythm of both parameters maintained the typical pattern, showing lower (“basal”) values during daytime (in the inactive phase of the day).

FGF21 increased the body temperature regardless of the presence of UCP1, both during the light and dark phases of the day (Figure 8C). In UCP1^+/+^ mice, FGF21 also increased energy expenditure (Figure 8F). However, importantly, induction of energy expenditure was completely absent in the UCP1-deficient mice (Figure 8F). The presence of UCP1 is thus required for FGF21-induced increases in energy expenditure – although its presence does not necessarily result in an FGF21-induced increase in energy expenditure (Figure 2). This experiment thus again reinforces different nature of FGF21 effects on body temperature and energy expenditure.

### 3.9 FGF21 reduces heat loss in UCP1-deficient mice

An important question relating to the above study is the mechanism for the FGF21-induced increase in body temperature in the UCP1-deficient mice, i.e. that an increase in body temperature occurs in the absence of an increase in energy expenditure. Body temperature is determined by the difference between heat production and heat loss. As heat production (energy expenditure) was not affected by FGF21 in these mice, we suspected that FGF21 treatment could reduce heat loss. Heat is dissipated through the body surface, especially through those parts that are not insulated by fur. A significant amount of heat can be dissipated via the tail [13,33,34]. Vasoconstriction of tail vessels can restrict heat loss. To assess whether this mechanism plays a role in increasing the FGF21-induced body temperature, the tail surface temperature was determined using an infrared camera throughout the FGF21 treatment.

First, we validated this technique in an extension of one of the previous experiments (Experiment 2), where FGF21-treated mice were injected with the β-adrenergic blocker propranolol and then with the β_3_-adrenergic agonist CL 316,243 (Figure 3G–H). The injection of propranolol led to a reduction of energy expenditure by 0.11 W and a reduction of body temperature by 0.65 °C (Figure 8G). As the tail temperature was not affected by propranolol (Figure 8H,I), we assume that the observed drop in energy expenditure was fully proportional to the decrease in body temperature. On the other hand, the injection of CL 316,243 resulted in a relatively significant rise in energy expenditure (by 0.18 W) but a relatively smaller rise in body temperature (0.47 °C; Figure 8G). Thus, it would be expected that the additional heat, produced after CL 316,243 injection, was dissipated. We observed a robust increase in tail temperature after injection of CL 316,243 (Figure 8H,I). Thus, the measurement of tail temperature seems to be a good indicator of heat loss management, providing data in accordance with the expectations. Notably, the tail temperature as such can be a very variable parameter, probably very much dependent on the individual conditions of the animal shortly before and during the measurement (Figure 8H). However, when the difference between tail temperature before and after the injection was calculated, the data became more homogeneous (Figure 8I).

Thus, we used this method also in the experiment with UCP1-deficient and control mice. We followed tail temperature in the days before the mice were transferred to the INCA chambers (Figure 8J,K). Just as in the pilot study, the tail surface temperature before the injection was very variable among the mice (not shown). Nonetheless, consistent data were obtained by calculating the difference between tail temperature before and after injection. In the UCP1^+/+^ mice, the FGF21 injection did not lead to systematic differences in tail temperature (Figure 8J). However, in the UCP1-deficient mice (that did not show any FGF21-induced increase in energy expenditure (Figure 8F)), the FGF21 treatment systematically decreased tail temperature (Figure 8K). Thus, FGF21 increases body temperature by decreasing heat loss, e.g. via the tail (at least in situations in which UCP1-dependent heat production is not available). This mechanism has earlier been demonstrated to influence body temperature in leptin-treated *ob/ob* mice [13] and in relation to different thyroid hormone-related effects [[34], [35], [36]].

## 4 Discussion

### 4.1 FGF21 has a pyrexic effect even in the absence of any increase in energy expenditure

In the present investigation, we demonstrate that, in contrast to the prevailing opinion, the FGF21-induced increase in body temperature is not a consequence of increased energy expenditure but rather a direct effect of FGF21 on the body temperature “setpoint” in the central nervous system. Thus, FGF21 causes pyrexia (or “fever”) in mice.

This conclusion is supported by several lines of evidence:

1. We observed a large FGF21-induced increase in energy expenditure in HFD-fed mice (Figure 3D, before CL) and only smaller (Figure 8D) or no induction (Figure 3, Figure 8B, before CL) in mice on the standard diet. This is principally in accordance with reports of varying magnitudes of metabolic effects of FGF21 among various mouse models [37]. In contrast, a robust increase in body temperature was observed in all conducted experiments. Thus, the increase in body temperature cannot be due to increased heat production, as it is observed even under conditions with no change of heat production.
2. The difference in body temperature between FGF21-treated and control animals is maintained even in a decreasing ambient temperature. Thus, the mice do not exploit the possibility that a lower ambient temperature provides in order to dissipate the “extra” heat, i.e. their elevated body temperature seems to reflect their body temperature setpoint rather than being increased secondarily to an increase in energy expenditure.
3. The elevated body temperature maintains a typical circadian pattern – higher during the night and lower during the inactive phase of the day, as is expected in pyrexic, not in hyperthermic animals.

The primary effect of FGF21 treatment is therefore a shift in the defended body temperature. The observation of a shift in body temperature is principally in accordance with earlier observations of a higher body temperature in mice overexpressing FGF21 [7] and a lower body temperature in FGF21-deficient animals with either total [7] or liver-specific FGF21 deficiency [38]. However, our interpretation of these changes is, in contrast to previous findings, that the body temperature effects are primary effects, not secondary to changes in energy expenditure.

### 4.2 An increase in energy expenditure by short-term FGF21 is mediated by UCP1

The elevated body temperature can be achieved by affecting the balance between heat production and heat loss. In UCP1-deficient mice, we find a reduction of tail surface temperature in response to FGF21, indicating a lower rate of heat loss. However, in wild-type mice, FGF21 induces UCP1-dependent energy expenditure. If UCP1 is activated, heat production increases and the elevated body temperature setpoint is thus achieved by these means, without any need for reduction of heat loss. Several studies have documented that FGF21 affects energy expenditure. However, these claims are mostly based on the so-called “divisor” problem: the misleading expression of energy expenditure per kg body weight in obesity-related research [[39], [40], [41], [42]]. Since FGF21-treated or FGF21-overexpressing mice (in contrast to their control counterparts) usually lose fat mass and body weight, the reported increase in energy expenditure can at least in part be attributed to this artificial calculation. For instance, in [5], an approximately 13–14% body weight reduction is brought about by FGF21 at a time when a 15–16% increase in energy expenditure (per kg body mass) occurs. This suggests that there is no change in energy expenditure when expressed per mouse (a more valid way of expression). Nevertheless, in several cases, the increased energy expenditure does not seem to be fully explainable by normalization to reduced body weight [2,3], and small but significant increases in energy expenditure are reported even when expressed per whole animal [4,6]. We confirm such an effect on energy expenditure in obese animals but we see rather limited (Figure 8D) or no induction of energy expenditure by short-term FGF21 treatment in chow-fed mice.

The FGF21-mediated elevation of energy expenditure has been attributed to the induction of UCP1. The mechanism of UCP1 induction, as well as its relative importance for whole-body energy expenditure, are still contested topics. FGF21 can increase UCP1 expression in cultured adipocytes [7] and 3T3-L1 cells [43], though a direct action of FGF21 on adipose tissue is not required for reduction of body weight since FGF21 lowers body weight equally well in animals with an adipose-specific deficiency in βKlotho (the FGF21 coreceptor critical for FGF21 action) [44].

Accumulating evidence suggests that most of the “thermogenic” effects of FGF21 are mediated by the central nervous system and adrenergic stimulation. We observe that acute administration of a β_3_-adrenergic agonist to HFD-fed mice treated with FGF21 has no additional stimulatory effect on energy expenditure. We conclude that the full potential for adrenergic induction of thermogenesis has already been brought about by the FGF21 treatment, i.e. that FGF21 acts mainly via activation of the sympathetic nervous system. Supporting this hypothesis, i.c.v. administration of FGF21 is sufficient to induce oxygen consumption and browning of WAT, while the β-adrenergic antagonist propranolol blocks these effects [45]. Here we demonstrate that propranolol attenuates even pyrexic and energy-expending effects of peripherally administered FGF21. Further, neither centrally nor peripherally administered FGF21 can induce UCP1 in β-less mice (mice lacking all isoforms of β-adrenergic receptors) [45]. In the same vein, subcutaneously delivered FGF21 fails to induce UCP1 expression and reduce body weight in a neuron-specific knockout of βKlotho [46]. Thus, although FGF21 may induce UCP1 gene expression directly in *in vitro* experiments, most of the physiological effects on energy expenditure and body weight are mediated via the central nervous system.

### 4.3 The significance of UCP1 for the effects of FGF21

The role of UCP1 activation in the body weight-lowering effects of FGF21 has been examined by studying FGF21 effects in UCP1-deficient mice [6,11]. However, the conclusions of those studies differed significantly. Both these studies showed that FGF21 lowered body weight regardless of the presence or absence of UCP1; this was corroborated by other studies as well [47,48]. Nevertheless, Veniant et al. [6] reported that FGF21 increased energy expenditure even in the absence of UCP1, while Samms et al. [11] observed that induction of energy expenditure was largely attenuated in UCP1-deficient mice (the body-weight lowering effect, in this case, maybe due partly to a decrease in food intake, as stated by Straub and Wolfrum [49]).

Thus, while Veniant [6] demonstrated that UCP1-deficient mice lose weight after FGF21 treatment due to a persistent increase in energy expenditure, Samms [11] showed that FGF21-treated UCP1-deficient mice lost body weight despite unchanged energy expenditure. This may be attributed to differences in the study protocols: the mice were housed either at thermoneutrality [6] or sub-thermoneutral temperature [11]; they were fed either standard chow [6] or HFD [11]; they were treated either with recombinant FGF21-Fc [6] (that may not cross the blood–brain barrier [50]) or with native FGF21 [11], and they received FGF21 for 16 days [6] or seven days [11].

Our results demonstrate that two days of FGF21 treatment can suffice to induce energy expenditure in wild-type mice (Figure 8D), but not in UCP1-deficient mice (Figure 8E). Thus, UCP1 is required for the semi-acute effects of FGF21 on energy expenditure. Also, energy expenditure increases gradually during more prolonged FGF21 treatment (Figure 6G,H), which is in line with several other studies [4,11,48]. Thus, significant effects of FGF21 are more frequently observed after longer treatment. Notably, although some energy-expending effects of FGF21 were reported in UCP1-deficient mice after long-term treatment, these effects were somewhat attenuated in comparison to UCP1 wild-type mice [6,48,49]. Hence, induction of energy expenditure is more probably observed in rodents with intact UCP1. Thus, FGF21 primarily increases energy expenditure by induction and activation of UCP1, and UCP1 is thus required for the full energy-expending potential of FGF21 to be manifest.

### 4.4 Central effects of FGF21

The temperature regulation centre is located in the preoptic area of the hypothalamus and affects several other hypothalamic areas controlling energy-saving and energy-expending effectors [51]. FGF21 has been shown to target several areas of the hypothalamus. In particular, the ventromedial hypothalamus and paraventricular nucleus were shown to play a role in mediating the suppression of carbohydrate intake [52] and a feeling of satiety [53], respectively. However, the central circuits mediating FGF21 effects on energy expenditure and body temperature have still to be characterized. Our results demonstrate that FGF21 elevates the body temperature setpoint, presumably by affecting the temperature regulation centre. We also demonstrate that induction of UCP1-dependent thermogenesis occurs in parallel (or with a slight delay) after the changes in body temperature (compare Figure 6C–D vs. Figure 6G-H, Figure 6I–J). Whether the activity of the hypothalamic temperature-regulating centre is solely responsible for the induction of UCP1 or whether sympathetic activation of BAT is triggered independently could not be ascertained in the current study, but we suggest that BAT activation is secondary to the increase in “setpoint”.

### 4.5 Induction of UCP1 in BAT vs. browning of WAT

The thermogenic capacity of adipose tissue is largely defined by the amount of UCP1 protein which can be activated by sympathetic stimulation. Sympathetic stimulation not only activates existing UCP1 but also recruits more UCP1 (via induction of UCP1 gene expression) in the long term. Thus, the important parameter to evaluate is the amount of UCP1 protein in the entire tissue (or, even better, in the whole animal). UCP1-mediated energy expenditure may be increased by sympathetic stimulation through activation of existing UCP1, even before the recruitment of new UCP1 becomes apparent. Therefore, the fact that we observed induction of energy expenditure (Figure 6E–H) while UCP1 amount in BAT was not significantly different (Figure 5H) does not demonstrate that the increase in energy expenditure is UCP1-independent.

Measurement of UCP1 gene expression is often used as a proxy indicator of BAT and WAT induction. It was, as noted, suggested that induction of UCP1 gene expression in white adipose tissue (i.e. browning) is the crucial process mediating the effects of FGF21 [7]. Thus, a robust relative induction of UCP1 mRNA by FGF21 was reported to occur in white adipose tissue (WAT), but not in brown adipose tissue (BAT): a 3-fold induction was found in BAT but 5-fold in WAT [5]; a 2-fold induction was found in BAT but a 20-fold induction in WAT [7]. That such changes in UCP1 mRNA do not result in observable amounts of UCP1 protein (as demonstrated here) is understandable because the level of UCP1 mRNA in WAT is very low, about 1000-fold lower than in BAT [20,26,54] (at thermoneutrality). Thus, even a 20-fold increase could not bring the UCP1 mRNA levels close to those in BAT. Given that protein amounts are largely determined by mRNA amounts (as they probably are [55]), the expected protein levels would still be below detection levels. Ultimately, it is the total amount of UCP1 protein (and not a fold-change in UCP1 gene expression) that defines the thermogenic capacity of the tissue [26]. Browning of WAT in connection with FGF21 treatment has received a lot of attention [6,7], while UCP1 amount in BAT has generally not been examined. We conclude that the hypothesis that FGF21 induces an increase in energy expenditure due to “browning” of WAT is not feasible: there is simply too little UCP1 protein in WAT. Rather, classical BAT would be responsible for any UCP1-dependent effect observed.

BAT is reported to be redundant for the effects of FGF21, as excision of interscapular BAT does not prevent the increase in energy expenditure and other effects [2,56]. However, if interscapular BAT is excised, compensatory recruitment of UCP1 in other depots can be expected, especially if animals face sub-thermoneutral temperatures [57,58]. BAT excision experiments are thus unable to exclude the role of UCP1 or the importance of BAT under physiological conditions. Therefore, the sum of UCP1 protein in BAT and WAT should be considered when assessing the capacity for non-shivering thermogenesis [50]. Here we demonstrate that, despite the FGF21 treatment, there is no detectable UCP1 protein in the inguinal WAT of lean or obese mice housed under human-like thermoneutral conditions, principally in agreement with [6]. On the other hand, FGF21 recruits UCP1-thermogenic capacity of BAT in lean, and especially in obese mice (Figure 3J). This result further emphasizes the need to assess UCP1 amount at the protein level (rather than mRNA) and, optimally, to calculate the amount of UCP1 per whole depot (rather than showing only UCP1/unit of protein).

### 4.6 Conclusion

The mechanism of induction of thermogenesis and body temperature by FGF21 is of crucial importance for understanding its body weight-lowering effects. Earlier studies have considered the increase in body temperature to be a consequence of increased energy expenditure. We provide a different point of view. Our results show that FGF21 raises body temperature independent of energy expenditure. In the absence of increased energy expenditure, elevated body temperature can be achieved by reducing heat loss. We conclude that FGF21 induces UCP1 in BAT, probably secondarily to the increased body temperature setpoint; in WAT, the level of UCP1 protein is too low to be detected. In contrast to earlier work [6,48], we demonstrate that activation of UCP1 is required for the induction of energy expenditure during short-term FGF21 treatment. Unless accompanied by increased energy expenditure (or reduced food intake), an increased body temperature cannot, per se, cause any reduction in body weight. Overall, our findings are of relevance in consideration of the use of FGF21 in the treatment of human obesity.

## Funding

The work was supported by 10.13039/501100001824Czech Science Foundation (19-05356Y) and the 10.13039/501100004359Swedish Research Council. The grant providers had no involvement in study design, data analysis, or interpretation.
